# Talar neck fracture combined with comminuted medial malleolar fracture: A case report

**DOI:** 10.1097/MD.0000000000045848

**Published:** 2025-11-07

**Authors:** Yuhang Zhang, Yimin Chen, Ke Zhou, Haitao Ma, Liming Zhu

**Affiliations:** a The First People’s Hospital of Xiaoshan District, Xiaoshan Affiliated Hospital of Wenzhou Medical University, Hangzhou, Zhejiang, China.

**Keywords:** AOFAS ankle-hindfoot scale, medial malleolar fracture, open reduction internal fixation, ORIF, talar neck fracture, transosseous tunnel sutures technique

## Abstract

**Rationale:**

A comminuted fracture of the medial malleolus combined with a talar neck fracture is a uncommon finding. Surgeons are often unaware of its unique injury mechanism, which complicates fracture reduction and fixation and may cause potential complications during and after surgery. This report presents a case of a talar neck fracture combined with a comminuted medial malleolar fracture and analyzes its injury mechanism.

**Patient concerns:**

A 35-year-old Chinese man sustained a talar neck fracture and comminuted medial malleolus in an electric bicycle accident, accompanied by inability to bear weight.

**Diagnoses:**

Radiographs and computed tomography confirmed a displaced talar neck fracture with intra-articular extension and a comminuted medial malleolar fracture. magnetic resonance imaging additionally revealed deltoid ligament rupture and tibialis posterior tendon exposure.

**Interventions:**

After 5 days of skin swelling resolution, he underwent open reduction and internal fixation using compression screws, Kirschner wires, and transosseous tunnel sutures.

**Outcomes:**

At the 10-month follow-up, radiographs showed satisfactory bone healing without avascular necrosis. Functional outcome improved significantly, with the American orthopaedic foot & ankle society ankle-hindfoot score rising from 20 preoperatively to 85 at final assessment.

**Lessons:**

This case supports pronation-dorsiflexion as the likely injury mechanism. Recognition of concomitant ligament and tendon injuries is critical. Combined screw fixation and transosseous tunnel sutures can achieve stable fixation, preserve ankle joint function, and reduce the risk of complications.

## 1. Introduction

Talar neck fractures are relatively rare injuries, typically caused by high-energy trauma, while medial malleolar fractures are commonly associated with rotational ankle injuries.^[1,2]^ In such complex injury mechanisms, not only should the talus be fixed to prevent ischemic necrosis, but stable fixation of the medial malleolus must also be considered to avoid secondary injury. The simultaneous occurrence of these fractures presents a significant challenge in surgical management and recovery. This case report presents a unique scenario of a combined talar neck and comminuted medial malleolar fracture in a patient following an electric bicycle accident and analyzes the mechanism of injury.

## 2. Case presentation

A 35-year-old Chinese male presented following a road traffic accident involving an electric bicycle. He reported severe pain and an inability to bear weight on his right ankle. There was no open wound, but significant swelling was observed. The patient’s ankle was immobilized with a plaster cast for 5 days to allow for swelling reduction before surgery. Surgery was planned as open reduction and internal fixation.

**Figure 1.**
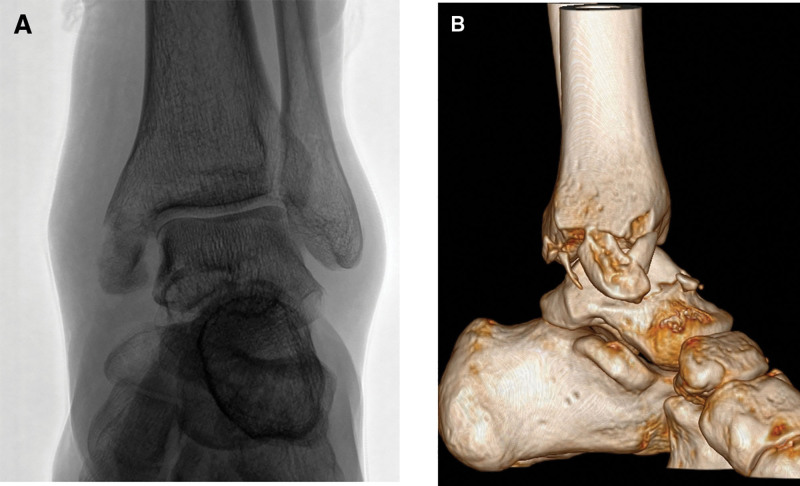
Preoperative imaging of the fractures. (A) X-ray showing a comminuted medial malleolar fracture. (B) Computed tomography scan revealing the comminuted medial malleolus fracture (including anterior and posterior fragments) and the talar neck fracture.

**Figure 2.**
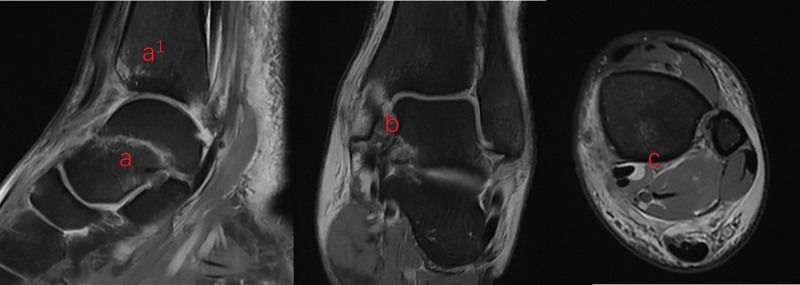
Figures A, A^1^, and B show the high signal of the talar neck, anterior malleolus, and medial malleolus respectively. Figure C show the extensive high signal in the tarsal tunnel.

Preoperative radiographs and a computed tomography scan (Fig. 1) revealed a talar neck fracture and comminuted medial malleolar fracture. The talar neck fracture involved a displaced fracture extending through the articular surface, while the medial malleolar fracture was highly comminuted, with fragments extending both posteriorly and anteriorly. Preoperative magnetic resonance imaging (MRI; Fig. 2) showed high signal intensity in the talar neck, anterior ankle, and medial malleolus. It is noteworthy that there was also extensive high signal intensity in the tarsal tunnel, while the lateral malleolus, lateral collateral ligament, and syndesmotic ligament did not show significant high signal intensity.

The patient underwent open reduction and internal fixation 5 days after admission. A standard medial incision was used to expose the medial malleolus. Due to the fracture, the talus was clearly exposed. After exposing the talar neck fracture line through the medial malleolus fracture line and achieving anatomical reduction, the talar neck fracture was fixed under fluoroscopic guidance. Two percutaneous screws were inserted from anterior to posterior to ensure appropriate alignment and compression across the fracture site, while percutaneous fixation preserves talar vascularity. The screws used were variable-diameter compression screws and a hollow compression screw, placed in a cross configuration to optimize stabilization.

**Figure 3.**
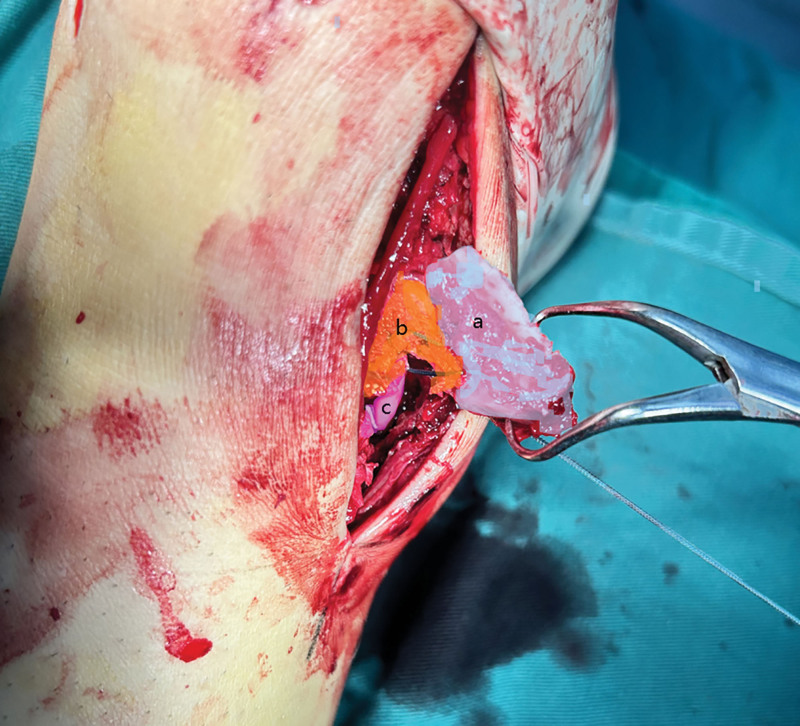
Intraoperative photos show that (A) main bone fragments of the medial malleolus; (B) damaged deltoid ligament and stump; (C) posterior tibial tendon. Deltoid ligament and posterior fracture fragment are bundled and fixed through suture tunnels.

**Figure 4.**
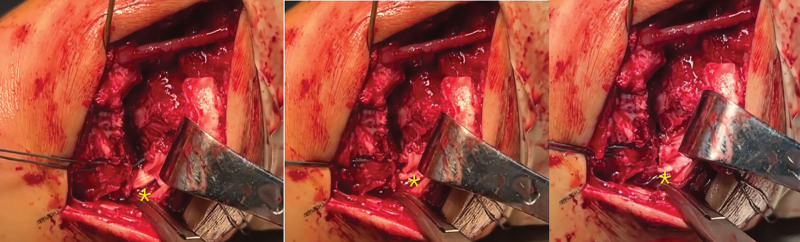
The * part of the picture shows the process of gradually tightening the sutures in the bone tunnel to restore the deltoid ligament and the posterior bone fragment, and the posterior tibial tendon is effectively covered.

**Figure 5.**
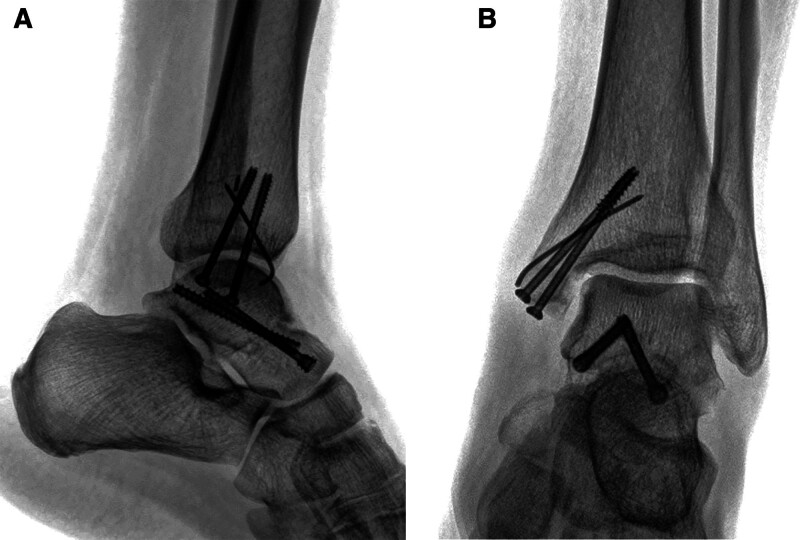
Intraoperative fluoroscopic views of the fracture fixation. (A) Image showing fixation of the talar neck and main medial malleolus fragments with cannulated screws. (B) Lateral view showing stabilization of the anterior crushed fragment with Kirschner wires. The broken end of the Kirschner wire remains within the tibia.

The medial malleolar fracture was more complex due to comminution. The anterior bone fragment of the medial malleolus was repositioned, and an additional 1.2 mm Kirschner wire was placed to fix the anterior fragment. Afterward, the main bone fragment was turned over to directly visualize the deltoid ligament, which was found to be torn in the posterior part and not connected to the posterior bone fragment. The exposed tibialis posterior tendon was also visible (Fig. 3). The transosseous technique was chosen for posterior fragments due to size (<5 mm) and ligamentous attachments, before repositioning and fixing the main bone fragment, Transosseous tunnels (2.0 mm drill) were placed in the posterior proximal tibia and nonabsorbable sutures were passed to secure the posterior fragment and deep deltoid ligament (Fig. 4). The main bone and posterior structure were then repositioned simultaneously, the main bone fragment was fixed with 2 cannulated screws (3.5 mm for medial malleolus), and the posterior structure was tightened and knotted with sutures. This was verified by intraoperative imaging. Interestingly, our intraoperative attempt to fix the main bone of the medial malleolus with smaller Kirschner wires failed, and the Kirschner wires broke, leaving fragments in the cancellous bone during removal (Fig. 5).

Postoperatively, the patient was placed in a splint and instructed to remain non-weight-bearing for 4 weeks. Sutures were removed after 2 weeks. At 1 month postoperatively, partial weight-bearing was initiated, and the patient progressed to full weight-bearing by the third month. The patient was followed for ten months postoperatively. Functional outcome was assessed using the American orthopaedic foot & ankle society ankle-hindfoot scale:

- Preoperative: 20;
- Postoperative week 1: 45;
- Postoperative month 1: 69;
- Postoperative month 3: 79;
- Postoperative month 10: 85.

**Figure 6.**
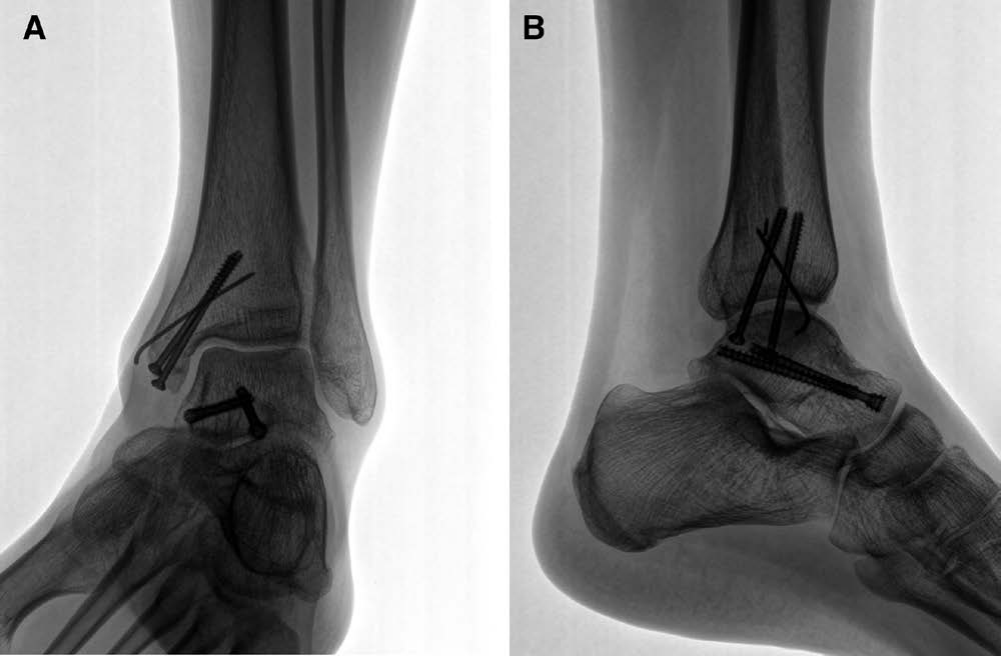
Postoperative follow-up radiographs showing fracture union. (A) Anteroposterior view revealing complete union of the talar neck and medial malleolar fractures. The presence of the Hawkins sign and absence of subchondral lucency indicate no evidence of avascular necrosis. (B) Lateral view confirming well-maintained ankle joint space without signs of early arthrosis..

Radiographs at follow-up confirmed adequate healing of both the talar neck and medial malleolus without evidence of avascular necrosis or malunion (Fig. 6).

## 3. Discussion

### 3.1. Characteristics of fractures

Talar neck fractures are a serious and relatively uncommon type of injury, accounting for only 0.14% to 0.32% of all fractures,^[3]^ and are often associated with high-energy dorsiflexion injuries.^[1,2]^ While medial malleolus fractures are common, the injury mechanism of comminuted medial malleolus fractures is complex and not associated with a specific type of injury.^[4]^ Cross-sectional studies have shown that talar neck fractures accompanied by medial malleolar fractures are relatively common.^[5]^ Hawkins found that 15 of 57 patients (26%), and Canale and Kelly found that 10 of 71 patients (14%) with talar neck fractures also had additional medial malleolar fractures.^[2]^ However, few publications describe the injury mechanism of talar neck fracture combined with medial malleolus fracture, and little attention is paid to the fact that this type of fracture is also complicated by medial ligament injury. While concomitant talar neck and medial malleolar fractures have been documented,^[6–9]^ this case uniquely demonstrates a comminuted medial malleolus fracture with deltoid ligament tear and tibialis posterior tendon exposure – a complex injury pattern rarely described in detail. This combination posed distinct surgical challenges requiring adaptive fixation techniques. In view of the various complications encountered during surgery in this case, the injury process of talar neck fractures and severe medial structural injury requires further study and analysis, and orthopedic surgeons should be aware of this in clinical practice.

### 3.2. Infer damage mechanism

**Figure 7.**
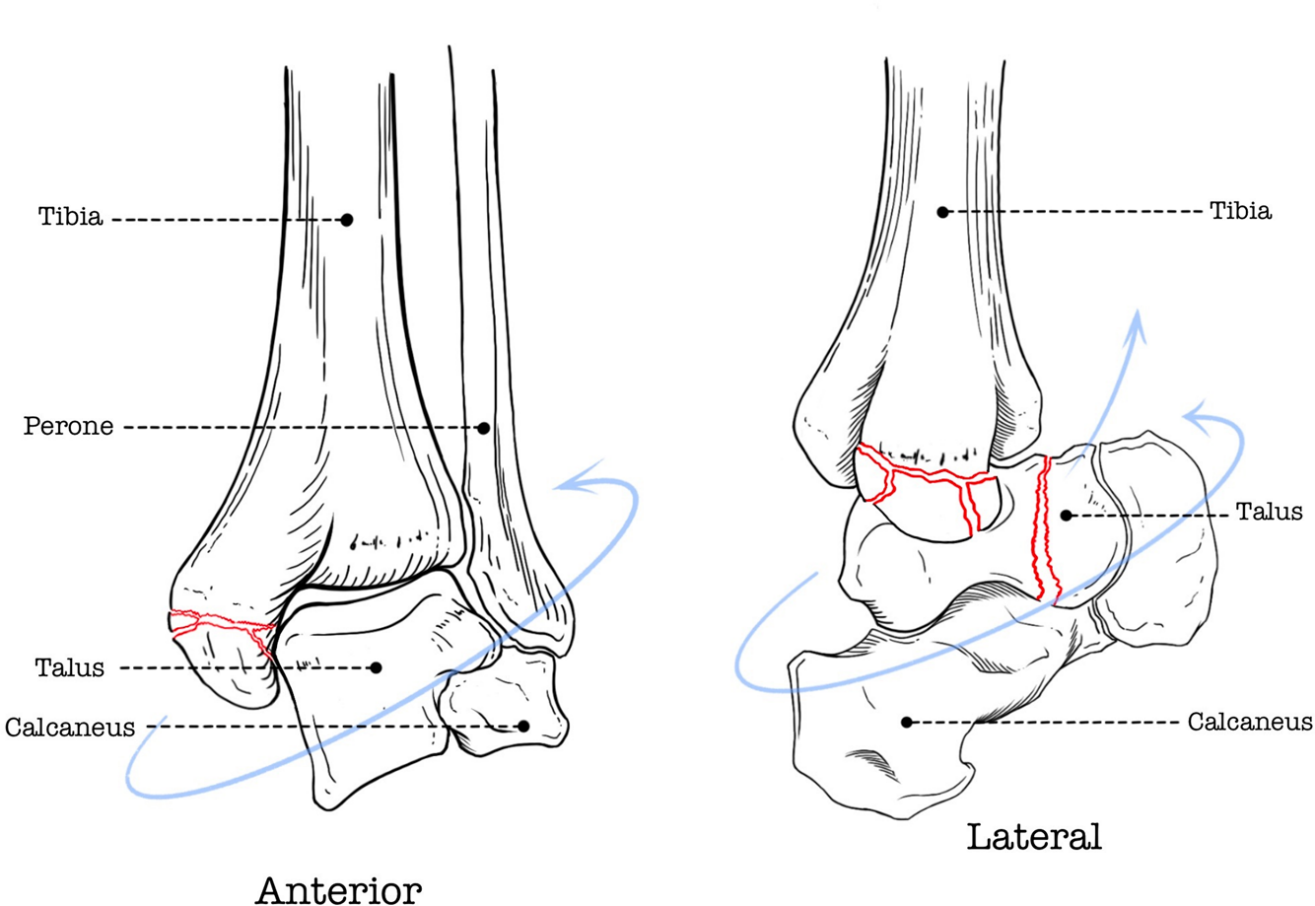
Schematic diagram of the forces that occur when a fracture occurs. First, the left diagram shows the mechanism of injury for a medial malleolus fracture caused by external rotation. The right diagram shows that there is a dorsiflexion force at the same time as the external rotation, which further leads to a talar neck fracture.

Based on fracture morphology (comminuted medial malleolus, intact lateral structures) and MRI findings, we hypothesize a pronation-dorsiflexion mechanism: forced dorsiflexion during pronation initially causes medial malleolar comminution, followed by talar neck impaction against the tibia. First, the medial malleolus and medial ligament are tense in the pronation state of the ankle joint, which is a prerequisite for medial malleolus fracture and medial ligament injury. This can be confirmed from the MRI cross-sectional images. The lateral structures of the patient’s ankle joint are not damaged (Fig. 2), which rules out supination injury. At the same time, due to the pronation and dorsiflexion of the ankle joint, on 1 hand, the medial malleolus is subjected to tensile stress from back to front, causing deltoid ligament tear and medial malleolus fracture to occur simultaneously. On the other hand, the talar neck and the anterior end of the tibia collide, resulting in a talar neck fracture (Fig. 7).

### 3.3. Treatment strategies

Fixation of the talus may be the easiest part of our surgery for this patient. In conventional treatment, the medial malleolus osteotomy approach is used to fully expose the talus.^[10]^ Fortunately, due to the comminuted fracture of the medial malleolus, the medial surface of the talus can be clearly exposed through its fracture line, which reduces the need for an additional osteotomy. Studies have shown that avascular necrosis occurs in 0.00% of type I fractures, nonsurgical treatment also achieves satisfactory results in adolescent talar neck fractures.^[11,12]^ However, it is still necessary to avoid iatrogenic aggravation of fracture displacement during surgery, as this may lead to avascular necrosis of the talus.^[13]^

The more challenging issue arises with medial malleolus fractures. Due to the irregular fracture pattern and potential soft tissue complications such as deltoid ligament involvement and tibialis posterior tendon dislocation, misdiagnosis is likely,^[14,15]^ which adds complexity to the surgery. Inadequate fixation can impair the stability of the ankle joint.^[16]^ In this case, we first fixed the comminuted anterior bone fragment. When exploring the posterior part of the medial malleolus, damage to the medial deltoid ligament and exposure of the posterior tibial tendon were found. The fracture fragment was smaller than expected before surgery, there was a lack of effective fixation methods. Our treatment plan included creating a bone tunnel at the proximal end of the fracture for suture passage. After passing the nonabsorbable suture, the distal suture was used to repair the damaged medial deltoid ligament, and the posterior fracture was sutured and fixed simultaneously. Unlike Isaacs et al^[7]^ (standard screw fixation) and Oesman et al^[17]^ (open reduction and internal fixation combined by Tension Band Wiring fixation) our case required transosseous tunnel sutures due to small posterior fragments and deltoid ligament tear – a scenario not previously addressed. The suture anchors were unavailable intraoperatively, and Kirschner wire fixation failed (Fig. 5). The transosseous technique was thus used to simultaneously fixate fragments and repair ligaments. While effective here, long-term studies are needed to validate its efficacy.

### 3.4. Highlights and flaws

The highlight of the case is the talar neck fracture and medial malleolus fracture combined with deltoid ligament injury, which is a less commonly reported type of injury. In the latest literature, both mild and severe injuries are reported: cases without talar fracture but with concurrent medial malleolus fracture and deltoid ligament injury; or severe injuries with talar (Hawkins 3) fracture and bilateral malleolus fracture.^[14,18]^ Ligament injuries often occur and are easily overlooked. Through imaging and anatomical analysis of the fracture morphology, we can roughly infer the process of this type of fracture, although this still requires further verification through biomechanical and cadaveric studies.

## 4. Conclusion

This case demonstrates the complexity of managing talar neck fractures combined with comminuted medial malleolar fractures. Surgical treatment using a combination of percutaneous screws, transosseous tunnel sutures, and Kirschner wires resulted in successful healing and functional recovery. The morphological analysis of the fracture injury in this case provides a basis for understanding the mechanism of this type of fracture. This mechanism remains theoretical; biomechanical studies are needed for validation. To better understand the optimal treatment strategy for this type of combined injury, future studies using cadaveric experiments may also be necessary to verify the mechanism of this type of fracture.

## Acknowledgments

We would like to thank Professors Dawei Bi and Jiakuan Ye for their invaluable assistance and support throughout this study.

## Author contributions

**Conceptualization:** Yimin Chen.

**Formal analysis:** Haitao Ma.

**Investigation:** Yuhang Zhang.

**Methodology:** Yuhang Zhang.

**Project administration:** Yuhang Zhang.

**Resources:** Yimin Chen, Liming Zhu.

**Supervision:** Haitao Ma.

**Validation:** Ke Zhou, Liming Zhu.

**Writing – original draft:** Ke Zhou.

**Writing – review & editing:** Yuhang Zhang, Liming Zhu.
